# Hospital Festivals, Meetings, &c.

**Published:** 1894-04-07

**Authors:** 


### HOSPITAL FESTIVALS, MEETINGS, ETC.

### THE ROYAL EYE HOSPITAL, SOUTHWARK.

The annual general meeting of the Royal Eye Hospital, Southwark, was held at the hospital on Wednesday last, Professor Malcolm McHardy, a vice-president and an honorary surgeon of the hospital, in the chair. The report of the council, and the accounts for the past year were received and adopted. In the first year from the opening of the new premises by the Duke of York, 13,221 new patients had been treated. Of these, 402 were in-patients. The hospital has accommodation for 40 in-patient beds, but of these, through lack of funds, 18 stand empty. The accounts showed that an additional income of fully £2,000 a year must be forthcoming to enable the institution to fully utilize advantages its new premises and special appliances are capable of affording. The new hospital's influence for good, which extended far and wide, had proved a timely and exemplary stimulus to other charities having the same object. This was evidenced by the determination to remove the ancient charity in Moorfields from its condemned premises and inappropriate site there to a modern building now in course of design by Mr. Keith D. Young, who was architect of the Royal Eye Hospital. Similarly, the Westminster Ophthalmic Hospital at Charing Cross was proclaiming the obvious inadequacy of its site and premises, and seeking £50,000 in consequence. It might turn out that a concentration of the in-patients of this and the last named charity at Southwark, and a remodelling of the out-patient department at Charing Cross, would be a mutually desirable, possible, and certainly economical solution of the matter. During the coming year sundry special efforts will be made to augment the at present gravely inadequate income; among these a generous performance by Miss Ellen Terry as Nance Oldfield, the valued co-operation of Miss Marion Kackenzie, a banquet and reception at the Grafton Galleries by the Lord Mayor and Lady Mayoress on May 25th, were mentioned; also a series of Cinderella dances under distinguished patronage, and a café chantant are being arranged for later in tne year.

### THE POPLAR HOSPITAL FOR ACCIDENTS.

The annual general meeting of the governors of this hospital was held on March 20th. The past year has seen considerable progress in the improvements and additions, and it is hoped that the whole hospital will be ready for use during the coming summer. The new wing was opened by Lord and Lady Knutsford in September last, and the urgent need for this hospital was abundantly shown by the fact that no less than thirty cases of accidents were admitted on the first day. But more funds are badly needed adequately to complete the work; especially is it desirable that the garden should be properly laid out for the use of patients, doctors, and nurses. Also another ward is much needed, and a new operating theatre, &c. Thoroughly to complete the hospital £3,000 is still required. The committee regret to report the death of Mrs. Jackson, who for twenty years held the post of matron at the Poplar Hospital, and to the last day of her life took the keenest interest in its welfare and progress. Miss Vacher, the present matron, has been compelled to take three months' rest, after a severe illness. The committee especially offer their thanks to her and to the nursing staff for their services, and for the cheerful way in which they have made the best of many difficulties and discomforts during the past year while building operations have been in full swing. Mr. Corner and Mr. Brownfield, who have held the posts of honorary surgeons for many years, intend to retire when the new building is fully opened, but will continue to help the hospital by serving on the committee.

